# “I Would Never Push a Vaccine on You”: A Qualitative Study of Social Norms and Pressure in Vaccine Behavior in the U.S.

**DOI:** 10.3390/vaccines10091402

**Published:** 2022-08-26

**Authors:** Cheryl Lin, Taylor Parker, Kartik Pejavara, Danielle Smith, Rungting Tu, Pikuei Tu

**Affiliations:** 1 Policy and Organizational Management Program, Duke University, Durham, NC 27705, USA; 2 Department of Business Administration, Tunghai University, Taichung 407224, Taiwan

**Keywords:** immunization, vaccine hesitancy, public health, health behavior, attitudes, perceptions, social factors, decision science, adoption, SARS-CoV-2

## Abstract

Previous researchers have established the influence of social norms on vaccine behavior. However, little work has been performed contextualizing individuals’ experience with these social factors and how they operate to persuade individuals’ acceptance or refusal of a vaccine. We aimed to determine the mechanisms of familial and societal pressure or expectations that contribute to COVID-19 vaccine decision-making. We conducted four focus groups and eleven individual interviews (total n = 32) with participants from across the U.S. of different vaccination statuses. We identified three emergent themes: (1) Altruistic reasoning was particularly prevalent among initially hesitant late adopters—the desire to protect loved ones and others constituted a dominant motive, more powerful than protecting oneself. Vaccination was also reckoned as part of a joint effort to return to normal life; hence, it invoked a sense of responsibility or “obligation”; (2) expectation often became pressure; although most vaccinated participants stated that they respected others’ choices, late adopters or unvaccinated participants perceived differently and felt rushed or “forced” into choosing, and many resented being “targeted” or “bullied”; (3) vaccination status became a new label, frequently dividing families, thus producing familial mandates, exclusions, or social stratifications. This caused sadness and feelings of isolation, along with the formation of a camaraderie among the excluded unvaccinated. A vaccine decision builds from the complexities of individuals’ experiences and cultures. The vaccinated were not free of hesitancy nor were the unvaccinated all anti-vaxxers. Vigorous vaccine promotion successfully converted some undecided individuals but also fostered distrust of government; alarmingly, the push to receive the COVID-19 vaccine further triggered doubts about established vaccines. Communication strategies need to be developed and implemented carefully so as not to ostracize the unvaccinated community and strengthen their resistance.

## 1. Introduction

The social, mental, economic, and public health consequences of the COVID-19 pandemic continue to be felt more than two years after its emergence. On the economic front, the lockdown and social distancing associated with COVID-19 led to pervasive disruption across sectors, including adverse impacts on transportation, retailing, and hospitality [1,2]. These impacts are also felt by the healthcare sector, which experienced a significant financial burden and surging demands for care, necessitating the development of a new vaccine or treatment [3]. However, the stress on the economy and on social and public health domains is still present, even with the release of effective vaccines [4]. This can be partially attributed to vaccine hesitancy [5,6,7], defined by the World Health Organization as a “delay in acceptance or refusal of vaccination despite availability of vaccination services [8].” Sixty percent of the U.S. population were fully vaccinated against COVID-19 by November 2021, but coverage slowed to barely top 67% by July 2022 [9]. Although many studies and polls have been conducted to determine individuals’ reasons to (not) obtain a vaccination [10,11,12], in-depth examination of the roles of social norms or pressure has been limited. Compared to the challenges of vaccine logistics or access, personal beliefs, including the fear of side effects and doubts concerning the necessity or efficacy of vaccines, might be amenable to change through encouragement via social factors. A greater understanding of inter-personal influences would benefit efforts to improve vaccine uptake by addressing persistent hesitancy.

Others’ influence on individuals’ behavior, widely discussed in the literature [13,14,15], also applies to vaccine-related decision-making [16,17]. This is particularly evident in the plethora of research on the importance of a physician recommendation, especially for the HPV and influenza vaccines [18,19,20]. Influence or motivation can come from outside of healthcare and distribution domains as well. Believing one’s friends and parents support the HPV vaccine was positively correlated with a higher intention to receive it [16]; for the influenza vaccine, individuals were more likely to get vaccinated if they thought others wanted them to obtain it [17]. Similarly, researchers found greater acceptance of the BCG vaccine among Nigerian mothers who lived in communities with pro-immunization activism [21]. On the other hand, nonconformists to recommended vaccination schedules have been associated with social networks that encourage such behavior [22], and low vaccination rates of homeschooled children could be explained by parents’ opinions of their friends’ vaccine hesitancy [23]. In the case of the measles outbreak of 2019, social media provided connections that allowed for the spread of “anti-vaccine” sentiments [24], and this homophily in personal or virtual networks can create echo chambers when online platforms provide users with information similar to their existing stance [25].

Further investigation of the influence of social factors concerning COVID-19 vaccines has been reported, including greater willingness to receive the vaccine when believing one’s friends and family support it and a positive correlation between vaccine intention and a perception that others would get vaccinated [26,27]. One study found that a person’s perceptions of the importance others place on COVID-19 vaccination were also associated with their own belief in the importance of vaccination and the assumption that more people were vaccinated [28]. Others have reported a relationship between prosocial behaviors and reduced vaccine hesitancy, especially in rural areas [29]. The effect of such assimilation or conformity can also be observed in other health behavior. Adherence to social distancing guidelines were best predicted by the perceived adherence of one’s close circle of friends and family [30]. However, not all scholars have concluded that social norms directly impact vaccination or preventive behavior. An experimental study derived that communicating favorable social norms had a weak link to increased vaccination intention and showed no difference than providing regular vaccine information [31]. Another noted having information about doctors’ vaccination behavior produced only a small positive effect on an individual’s own decision to be vaccinated [27].

SARS-CoV-2 (severe acute respiratory syndrome coronavirus 2) has demonstrated the ability to spread rapidly and quickly mutate, thus rendering the achievement and maintenance of high vaccine coverage of critical importance. We aimed to add to the here-to-fore mostly quantitative nature of the literature by determining the mechanisms of social norms in contributing to COVID-19 vaccine decision-making. Our objective was to contextualize the relationships with descriptive accounts of personal experiences. We chose a qualitative analysis to provide a richer insight into the prevalent social factors and perceptions to examine the following research questions: whether and how others’ opinions or societal expectations help overcome vaccine hesitancy, how a self-prescribed social role influenced behavior change, and how these elements combine to encourage vaccine acceptance or its refusal. We were especially interested in the channels or motivations that converted those who were initially hesitant or undecided but later chose to obtain a vaccine in order to help inform how to more effectively encourage acceptance among those still unvaccinated. In the follow sections, we depict the research design and data collection, synthesize the findings with illustrative quotes from participants, and discuss the results and implications, including a summary table presenting the contribution to the field. Our results could also help explain existing contradictions in the literature and address the gap concerning the effects of social pressure on an individual’s choice to obtain a COVID-19 vaccine.

## 2. Methods

### 2.1. Participants and Data Collection

Our qualitative research comprised 4 90-min focus groups (FG, each with 4–6 individuals) and 11 60-min in-depth individual interviews (IDIs). To obtain a wide spectrum of perspectives and experiences, we considered individuals 18 and older, living in the U.S., with any vaccination status to be eligible, and we made a particular effort to oversample racial minorities. Participants were recruited through personal networks, posting flyers, and social media (Reddit, Facebook, and Instagram) to reach potential participants in all regions of the country. The research was approved by Duke University Institutional Review Board. To encourage candid opinion sharing and avoid hostile interactions due to dissimilar views, we screened interested respondents and then sorted them so that all participants within each FG had the same vaccination status:

FG-A: vaccinated late adaptors, defined as receiving their first dose of the COVID-19 vaccine on or after May 2021, all of whom also received a single booster shot.

FG-B: vaccinated late adopters who had not received a COVID-19 booster shot.

FG-C: unvaccinated individuals.

FG-D: vaccinated early adopters, defined as receiving the first dose of the COVID-19 vaccine before May 2021, only some of whom had received a booster shot.

The discussion grouping design was intended to cover diverse vaccine attitudes and positions. Early adopters were generally pro-vaccine and signed up for vaccination appointments as soon as possible for them; late adopters often had (or still have) a certain level of hesitancy but accepted becoming vaccinated for reasons that could inform strategies to persuade non-adopters. We chose May 2021 as the cut-off time for adoption categorization because it was six months after the FDA approved two COVID-19 vaccines for emergency use in December 2020 in the U.S. and more than one month since the entire U.S. population was eligible to be vaccinated. Along with focus groups, separate IDIs were conducted to explore thoughts and feelings in further detail. In total, 32 people were included in the study: 21 individuals participated across 4 FGs and another 11 individuals took part in IDIs. Informed consent was obtained prior to each session.

A semi-structured discussion guide was created for both the FGs and the IDIs. It covered multiple areas, including participants’ thoughts and feelings about the COVID-19 vaccine and vaccines in general, pandemic-related experiences and emotions, and factors that may have influenced their vaccination decision such as availability, concerns, perceived norms, promotional communications or requirements (e.g., mandates, recommendation), and views on people of a different vaccination status, with a particular focus on social and interpersonal relationships and interactions. A trained moderator conducted all FGs, and two research team members conducted the IDIs. All sessions were video recorded and transcribed by two team members, with a minimum of two passes conducted for a quality check.

### 2.2. Data Analysis

We used NVivo 12 in our qualitative data analysis to code the FG and IDI transcriptions. NVivo 12 allows for organization and management of qualitative data through the creation of nodes (categories and codes) into which selected text data can be placed [32]. The software comprises a search function for efficient identification of key information and common themes [32]. This facilitates ease of retrieval and visual organization of the qualitative data [33].

The codebook was first conceptualized deductively based on the sub-topics covered in the discussion guide; it was then revised by the research team after reviewing and test-coding the transcriptions to better capture and categorize the text data. The final codebook centered on relevant vaccination matters, including vaccine hesitancy, information participants sought or received, their trust in healthcare and the healthcare system, and the influences of social factors on their decision-making processes. Two research team members independently coded each session, and disagreements were resolved through team deliberation. We employed an inductive, thematic content appraisal approach for our analysis [34]; categories of social factors and behavior were identified via iterative discussions of recurring participant reflections, and emerging themes were decided through consensus among research team members.

## 3. Results

### 3.1. Participant Characteristics

Of our 32 participants, slightly more than half were female (n = 17, or 53.1%); 87.5% identified as part of a racial minority group (40.6% Black, 28.1% Hispanic, 15.6% Asian, and 3.1% Native American) and the remainder were White (12.5%). The average age was 38.7 years old (SD = 11.1). The majority of our sample population was highly educated: 15 (46.9%) had a 4-year college degree and 9 (28.1%) had completed graduate studies. Twenty-three (71.9%) of the participants were vaccinated, of whom more than half (58.3%) were late adopters by our definition.

We identified three primary emerging themes related to the social and interpersonal domains contributing to participants’ behavior or position on the COVID-19 vaccine: (1) the strong desire to protect those around oneself, which might extend to the larger community; (2) varied forms of social pressure intended to induce vaccine uptake, sometimes to the participant’s subsequent resentment; and (3) social stratification between vaccinated and unvaccinated individuals.

### 3.2. Prominent Motive of Protecting Others

#### 3.2.1. Safeguarding Family and Friends

Among the vaccinated participants, many decided to obtain the vaccine predominately to protect those they cared about, often viewing this reason of equal or greater importance than protecting themselves. This attitude was particularly conveyed by late adopters who were initially reluctant about obtaining a vaccination, either due to not perceiving a need for it or worries about side effects:

“*I actually talked to my family first … I was the one that was traveling more and I was around people more … so I did it mainly because [of] them because I’m out and I don’t want to expose them based on me*”

—(FG#A-5)

“*My parents are in their mid to late 70s. And so I think about not that it’ s going to prevent me necessarily from getting COVID, but I would hope that it would prevent me from even passing it to them.*”

—(FG#A-4)

Further, this protective intention was not just about preventing loved ones from being exposed to COVID-19 but also due to fear of the participant contracting the disease and thus not being able to perform the caregiver role:

“*I want to go ahead and do it because I prefer to have a shot and not to be sick. Because I got my family, I got my kids, and I want to be okay for my kids and my husband*”

—(IDI#5)

#### 3.2.2. Protecting Vulnerable Individuals and the Community

Beyond protecting family and friends in general, a number of participants noted a specific emphasis on shielding vulnerable individuals (i.e., young children, the elderly, immunocompromised individuals, or those undergoing cancer treatment). Becoming vaccinated, participants felt, provided them comfort when spending time with these individuals as they were less likely to put them at risk of contracting COVID-19:

“*I remember getting my vaccine and just feeling like, oh okay, hope, I have hope again. You know, that my little baby we wouldn’t accidentally give him COVID*”

—(IDI#3)

Participants also discussed the desire to help their immediate community or the greater society, including people they did not know personally. This altruism was raised by more than half of the participants. Moreover, several minority participants mentioned that feeling the need or the expectation to protect vulnerable groups was likely due to cultural values (e.g., respecting the elders and caring for the weak):

“*I think that my students were [a] very big motivation to me because they were young and they were not getting any kind of [protection] and they also have families, and some of them are being raised by grandparents… it was a no brainer [to get the vaccine].*”

—(IDI#10)

“*A tremendous part of the influence on me was um everyone else, know keeping everyone else safe, our baby, the elders. How do we keep everyone in this community safe? Vaccines do that.*”

—(IDI#1)

#### 3.2.3. Vaccination As a Responsibility and Joint Effort

A good number of participants, with attitudes either favoring vaccines or neutral concerning them, described vaccination as a responsibility and a means to return to normal. Participants believed in contributing to the well-being of the broader population; they sometimes cited concepts of morality in their vaccine-related discussions, e.g., referring to becoming vaccinated as the “right” decision or even as an “obligation”:

“*I always think about the small kids …and friends that work in the hospital. And I just saw what this virus has done to them and it was one of those things that I felt like I had to do my part and get the vaccine.*”

—(FG#A-3)

“*I feel like at least from my friends who are East Asian they just kind of accept it as like a collective thing to do, not only for themselves, but for the community, um that kind of is embedded in the culture*”

—(IDI#11)

Many of both early and late adopters further expressed frustration with why some people would not accept the vaccine:

“*People just don’t understand the basic science, or the ability to actually help your fellow man, like making a slight sacrifice, by getting a needle in you.*”

—(FG#B-4)

“*You’re privileged enough to be able to have the vaccine to protect yourself and other people… I’m not saying that freedom is not important, of course you have the right to choose stuff for your body. But like, don’t be so high off it that you’re totally impacting other people, and you’re not really caring for yourself, you know.*”

—(FG#B-1)

At the same time, there was an unexpected and touching sense of togetherness participants experienced from the act of vaccination. This shared behavior was uplifting for many in the depressing and “crazy” COVID-19 pandemic atmosphere:

“*I have seen a sense of unity among this country and almost worldwide that I haven’t seen in a very long time. So, um that’s one of the greatest benefits because people who were very independent on it, they are looking for harmony to resolve something, even with the social distancing.*”

—(FG#A-6)

“*While I was at the pharmacy, a couple of other people came for [the] vaccine. So, it just made me feel good to know that at least some people out there are doing what they should be doing.*”

—(FG#B-4)

### 3.3. The Attributes and Consequences of Social Pressure

Participants also described how they communicated their beliefs to those around them and how they interpreted the messages they received. Some were active in persuading hesitant individuals to become vaccinated. Participants reported two levels of social pressure: indirect (where people the participant knew announced their vaccination status, with the implication that the participant should also become vaccinated) and direct (where people explicitly told the participant to become vaccinated).

#### 3.3.1. Pressure and Persuasion from Vaccinated Individuals

Most vaccinated participants believed that others ought to be vaccinated. At the same time, many added that they would still respect individuals’ decisions, even when these decisions contradicted with the desired choices. However, unvaccinated participants interpreted the messages differently and experienced tension:

“*So it’s one of those things that I just, I explained to them. Yes, I got it. And you know, I didn’t have any side effects—thank God. And that I just see the benefit in it, but I would never push that on you. That’s your choice.*”

—(FG#A-3)

“*I got a few of my friends, I have them go get vaccinated. I will still do that if I’d be making sense to some people… we try to keep it like uh, we don’t force you.*”

—(IDI#2)

Others took stronger approaches. One minority participant described how the members of their community frequently suggested that others become vaccinated, making being vaccinated appear to be “the norm” in their community:

“*My aunt, cousin, and my great aunt … had polio as a child, and they were loud at the beginning of this [pandemic]. They were like ‘you’re getting vaccinated, right? You know there’s vaccine clinic, right?’ I mean they were loud in everybody’s ears, because they had to limp in the wheelchair.*”

—(IDI#1)

“*I would probably just ask about even if you don’t want to do it for yourself, do you want to do it for the people that are around you and the people that you come in contact with?*”

—(FG#D-1)

#### 3.3.2. Feelings of Resentment Stemmed from Social Pressures

Unvaccinated individuals and some late adopters developed negative feelings from surrounding social or personal pressures. A few disclosed that their family had outcasted them, which upset them. Some participants became “immune” to the persistent push:

“*[The pressure] was from family. And it did hurt. It hurt that they would try to pressure me into getting the vaccine.*”

—(FG#C-5)

“*My family and friends say a lot [about the vaccine safety]… ’oh it don’t do anything to you, I haven’t felt anything [side effects] in months.’ But it still doesn’t influence me in any type of way.*”

—(FG#C-4)

Campaigns surrounding vaccine availability, the initial excitement surrounding the release of the vaccine, and the eventual vaccine requirements of some businesses all resulted in feelings of tension or annoyance among unvaccinated participants. Even some vaccinated participants found the ever-present promotion irritating:

“*It started becoming almost like a trend on social media, like Instagram. I remember seeing when it [the vaccine] first came out, people taking pictures of their vaccination cards. And like, ‘I got my vaccine.’ It was like a ’Oh my gosh!’. Like the golden ticket. I got it. But to me, I was like, go ahead and keep that golden ticket.*”

—(FG#A-2)

“*I also feel the pressure from these businesses that say you can’t go in there unless you have your vaccine card. And so… honestly, I don’t think it’s fair.*”

—(FG#C-4)

Many unvaccinated participants were quick to declare that they were not anti-vaxxers; they were just taking their time with the COVID-19 vaccine or had not seen sufficient evidence of its benefit. A few questioned why the vaccine was “needed” if even the vaccinated could still become infected. They resented being “targeted” or “bullied”, which “caused [them] a lot of anxiety and depression”:

“*I think they are exploiting the opportunity or the weakness…I don’t like that so, I don’t want to be pressured into making decisions that I would later regret.*”

—(FG#C-2)

“*If you feel the pressure, if you feel that you think you need it, you should go get it [the vaccine], I’m not stopping you. All I’m asking is don’t force me to do what I don’t want to do.*”

—(FG#C-1)

“*So, the pressure from the media, it put me off…that’s just kinda woahhh wait a minute, you’re rushing me.*”

—(FG3#C-6)

#### 3.3.3. Forced Vaccination Corrodes Trust and Support

Several unvaccinated and late-adopter participants shared the feeling that institutional entities were trying to force them to vaccinate. Many late adopters only obtained the vaccine because their work required it. These participants disliked the government’s strongarmed approach to vaccinating the U.S. population, and some lost faith in governmental authority because of this practice:

“*I had to do it for work, so I was like, okay, what else or why else would we be doing this right now in this need without a lot of experimentation on it?*”

—(FG#A-5)

“*At first I was scared, and was about to get the vaccine, but I was like that’s what the government wants you to do. They want you to get scared and go and get a vaccine. And so, instead of going, I stayed at my house for the longest [time].*”

—(FG#C-4)

Some of the initially undecided or still unvaccinated participants wondered if there was a hidden agenda behind the vigorous promotion (e.g., “a monetary gain”). They even started to question other previously received vaccines:

“*Well, I have children... and I just throughout their youth, I assumed that the shots offered at the doctor’s office and required by the school system were tested and safe... I began doing some more research and started feeling a little fishy about even the established vaccinations. I want more proof… maybe there’s nothing in there. Maybe it’s just a- an empty vial they’re making money off of. I question everything now.*”

—(FG#C-6)

### 3.4. Social Stratification of Groups

#### 3.4.1. Familial Mandates and Separations

Amidst ongoing and heated discourse on vaccines, participants experienced social stratification based on their vaccination status. Participants described how some vaccinated individuals threatened the unvaccinated with social exclusion:

“*For me it was mandated by my dad and my mom. I couldn’t go home to see them if I didn’t get it [the vaccine]… I actually had family to fallout behind it. Um between quarantine and the vaccine, they had to move out of the house because they wouldn’t get it.*”

—(FG#A-1)

“*My mother is in her 90s, and so to go see her, my sisters were trying to pressure me into getting the shots.*”

—(FG#C-6)

“*Well, it made me feel sad because uh we love to barbecue, and we find any reason to have a barbecue. And there’s a bunch of us and now they don’t come over… Um, they’re all vaccinated.*”

—(FG#C-5)

#### 3.4.2. Camaraderie among the Unvaccinated

Many unvaccinated participants self-identified as part of a larger unvaccinated community:

“*Definitely, I feel more at ease with these [unvaccinated] people because they are ‘crazy’ like I am.*”

—(IDI#9)

During their 90-min FG, a bond was formed among the 6 unvaccinated participants, despite their different attitudes towards and reasons for not accepting the COVID-19 vaccine, which ranged from outright objecting to vaccines or disagreeing with the mandate, to being open to considering additional evidence. They were connected by their unvaccinated status, developed a sense of camaraderie, and shared accounts of being discriminated against or pressured to become vaccinated. One participant stated that she would be willing to go visit some of her FG peers who had been excluded by their families:

“*I say we’ll be your family now. I’ll come see [participant 4] and I’ll- I love Texas. [Participant 5], I’ll come see you.*”

—(FG#C-6)

A closeness or immediate understanding between unvaccinated individuals was apparent. We did not observe this sense of community in the FGs of vaccinated participants.

**Figure 1:**
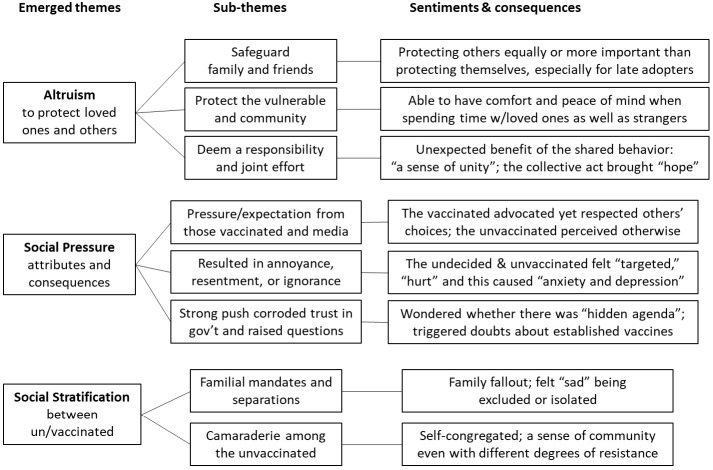
The influence of social norms and pressure on COVID-19 vaccine behavior.

Figure 1 synthesizes the themes that emerged relating to social factors and the sentiments and consequences that stemmed from them.

## 4. Discussion

This qualitative study with participants of various vaccination statuses portrayed how familial motivation, norms, and social pressure could influence decision-making concerning COVID-19 vaccines. Our analysis suggested these interpersonal factors and expectations, whether explicitly stated or self-perceived, could act as encouragement or upset and further discourage those unwilling to become vaccinated.

Notably, vaccinated participants were not all pro-vaccine or free of hesitancy. It is important and prudent to explore how and why initially reluctant individuals had accepted the vaccine rather than simply contemplating the reasons for which the unvaccinated continue to reject vaccines. Our research design separated early and late adopters during data collection to discover and distinguish these insights, so the findings could potentially inform communications to encourage vaccination among those hesitant or persistently opposed to becoming vaccinated. The literature indicates people’s motivation for change frequently results from the desire to protect themselves, friends, and family [35,36,37], with altruism reported to positively influence vaccination [38,39]. Our findings add to this by identifying altruism as a prominent driver among late adopters who may still have doubts but chose to do “the right thing”. Vaccination afforded our participants comfort and peace of mind when spending time with loved ones, as they knew they had reduced their risk of transmission. We also observed that the sense of responsibility extended to the greater community, including strangers. Participants stated that, although they obtained their vaccines under different levels of willingness or confidence, performing and witnessing this shared action instilled hope beyond the acknowledged efficacy of the vaccine.

In the individual and group discussions, participants provided accounts about and reflections on the ways they experienced direct and indirect social pressure; these are details not offered in the extant COVID-19 vaccine decision literature. Although early adopters were more likely to trust science, late adopters could also become advocates after they recognized the vaccine’s benefits. Both groups shared approaches to motivate those undecided or rejecting vaccination, including family and friend recommendation, modeling, and push. According to the Survey Center on American Life, individuals are more likely to obtain vaccination if they have mostly vaccinated friends [40]. This correlation can also explain why participants from tightly knit Hispanic, Asian, or Native American communities saw high vaccine uptake. Culture has a complex role in vaccine decision-making, as evidenced in previous research [41,42], and participants in our study reported their cultural values either instilled beliefs or shaped norms promoting vaccination, even while they remained doubtful of the vaccine.

Vaccinated participants have provided various forms of vaccine advocacy. Though they stated that they respected others’ opinions and decisions, those who were unvaccinated often perceived otherwise, reporting social pressures that induced feelings of antipathy, sadness, and bitterness. Thus, a poorly worded or overly persistent message could create the opposite effect from that intended [43]. Additionally, vaccinated participants, especially minorities, felt offended by some vaccine-related messages and efforts in their community that referenced the relationship between racial minorities and high COVID-19 infection or mortality and low vaccination rates. Moreover, participants’ trust in the government decreased due to the forceful nature of vaccine messaging. This further harms vaccine uptake as an individual’s willingness to vaccinate has been shown to be correlated with their level of trust in the government [44]. While the vaccinated viewed vaccination as the responsible thing to do, the unvaccinated considered their freedom of choice as more important. Mandates might be perceived as a threat to autonomy, leading to further distrust [45]; such sentiment could also harm the acceptance of boosters.

The descriptions of social and self-isolation emerged repeatedly in conversations with participants. Polarization in vaccine attitudes was a primary reason for the separation of unvaccinated participants or late adopters (before they received the vaccine) from their families. Vaccination status became a label and a new categorization. Many unvaccinated participants reported that vaccinated family and friends would not socialize with them. Even for vaccinated participants, some reported becoming vaccinated only to address “mandates” from family. A poll indicated one-third of vaccinated U.S. residents had cut connections with unvaccinated friends and family members [46], and half of them were uncomfortable spending holidays with unvaccinated individuals [47,48]. The communal stance against being “ostracized” by the vaccinated community may make the unvaccinated population more extreme in their viewpoints and more resistant to interventions.

Table 1 compares the existing literature with our findings and presents the contribution this study adds to the field.

Although we generated rich insights due to the strategies we used to recruit a diverse sample, the qualitative nature of our work inherently presented certain limitations, including potential bias in coding and interpreting the data. We attempted to minimize subjectivity by having multiple researchers review and code the transcripts and iteratively evaluate emerging themes. The research design separating the vaccinated from the unvaccinated in data collection provided a safer environment for participants to candidly share their views within the respective focus groups. At the same time, this limited opportunities to observe interactions or debates between people of opposite stands. Furthermore, as we conducted our interviews online, a lack of Internet access might have excluded certain groups. Participants may have felt less comfortable sharing by video connection as opposed to in-person. We observed, however, that participants warmed up quickly as the conversations progressed.

### Implications for Practice and Future Research

Although attempts by institutions to have more people vaccinated (including mandates) have experienced some success, these impersonal efforts have also produced resistance, resentment, and distrust of government. The seemingly relentless push has alarmingly triggered doubts about well-established vaccines. The spill-over effect of confidence corrosion in vaccines and the healthcare system calls for attention in order to secure high vaccination rates for the COVID-19 booster and protect the population from other diseases as well. Future studies could conduct longitudinal surveys to examine the impact of mandate on initial vaccination acceptance compared to subsequent voluntary booster uptake.

Furthermore, the effectiveness of educational campaigns concerning the vaccine’s purpose and risks associated with the virus was reflected in participants’ assertions of caring for vulnerable groups and the importance of collectively achieving a high vaccination rate. Nevertheless, the fact that some disputed the vaccine’s necessity or efficacy (i.e., vaccinated people can still become infected) indicated more clarification of expectations is required.

Extended research could further analyze how influential social elements are in relation to other factors that may motivate behavior change concerning vaccine acceptance. With resentment and stratification emerging as crude answers to the discomfort felt by individuals with different vaccine positions, it is critically important to further evaluate these phenomena and methods to more sensitively frame messages intended to increase vaccination rates.

## 5. Conclusions

There are multiple social factors at play in the decision to obtain or to reject the COVID-19 vaccine, with altruism, social pressures, and social stratification emerging as the three most common themes in our study. Altruism was particularly prevalent among late adopters, and for this same group, vaccination was perceived as a responsibility. This sense of a shared responsibility to obtain vaccination among those vaccinated often inadvertently turned into unwelcomed pressure for the unvaccinated. In combination with this pressure, vaccination status became its own label, creating tension and strife between family members of different statuses and feelings of isolation for unvaccinated individuals. In pursuing interventions to reach the unvaccinated community, it is vital not to marginalize their concerns due to the resentment that can result. A compromise must be found that incentivizes vaccination without antagonizing or isolating unvaccinated individuals. Those individuals who remain unvaccinated may be motivated to change their status if the inducement is reducing the risk of infection for vulnerable others, or if the choice is seen as honorable rather than forced.

## Figures and Tables

**Table 1:** Summary of the current study contribution compared to existing research on social norms and influences.

Existing Work	Our Results	Contribution to Literature
Many people listed the desire to protect those they care about as a primary motivator for vaccination [35,36,37,38,39].	Participants felt more comfortable meeting loved ones after vaccinating; the intention to protect others extends to include the greater community and people they do not know. Late adopters felt they were acting in good faith by becoming vaccinated.	The decision to vaccinate was felt as a moral obligation, which especially motivated those originally hesitant. The desire to protect the vulnerable groups and the public from the harms of the virus can be tied to the success of educational messaging on COVID-19.
Individuals are more likely to vaccinate if a large portion of those they know are vaccinated [40]. Culture and community have been shown to influence COVID-19 vaccine uptake [41,42].	Participants from close-knit communities saw high vaccination rates in their communities. Social norms were shaped around positive vaccination status, regardless of one’s individual beliefs.	Our study reaffirms the findings that communal and cultural beliefs have a strong influence on vaccine uptake. It adds that some institutions using stringent tactics have conversely fostered greater resistance to vaccinate.
In many scenarios, over-persistent messaging has led to increased feelings of hesitancy toward the COVID-19 vaccine [43]. This, in addition to more forceful efforts, has been detrimental to COVID-19 vaccine uptake [44,45].	Many vaccinated minorities felt offended by the messaging about vaccine uptake being low in their communities and the types of efforts used to increase uptake. “Targeted” promotion could generate resentment and feelings of being bullied.	The results provided evidence that improper messaging could jeopardize public perception of the COVID-19 vaccine, which could hurt vaccine acceptance.
Many U.S. residents have been avoiding unvaccinated friends and family [31], even refusing to spend holidays with them [32,33].	Many unvaccinated participants have been rejected by their families because of their vaccination status, and some vaccinated participants felt obligated to vaccinate to avoid being excluded or isolated.	Ostracization by friends and family has been effective in some cases, but it has also increased polarization and resistance to vaccinate in some.

## Data Availability

A portion of the deidentified data directly related to this paper is available from the corresponding author one year from the data of publication with reasonable and sound research proposal.
